# Gambogic acid suppresses cancer invasion and migration by inhibiting TGFβ1-induced epithelial-to-mesenchymal transition

**DOI:** 10.18632/oncotarget.15449

**Published:** 2017-02-17

**Authors:** Kai Zhao, Shuai Zhang, Xiuming Song, Yuyuan Yao, Yuxin Zhou, Qidong You, Qinglong Guo, Na Lu

**Affiliations:** ^1^ State Key Laboratory of Natural Medicines, College of Basic Medicine and Clinical Pharmacy, China Pharmaceutical University, Nanjing 210009, People's Republic of China; ^2^ Department of Thoracic Surgery, Nanjing Medical University Affiliated Cancer Hospital, Jiangsu Key Laboratory of Molecular and Translational Cancer Research, Cancer Institute of Jiangsu Province, Nanjing 210009, People's Republic of China; ^3^ Chia Tai Tianqing Pharmaceutical Group Co., Ltd, People's Republic of China

**Keywords:** gambogic acid, invasion, A549 cells, EMT, NF-κB

## Abstract

The epithelial-to-mesenchymal transition (EMT) contributes to the disruption of cell–cell junctions and imbues cancer cells with invasive and migratory properties. In this study, we investigated the effect of gambogic acid, a xanthone extracted from the resin of Garciania hanburyi, on transforming growth factor β1 (TGFβ1)-induced EMT. Gambogic acid inhibited the invasion and migration of TGFβ1-induced A549 cells *in vitro*. Gambogic acid also increased the mRNA and protein expression of E-cadherin, but repressed the mRNA and protein expression of N-cadherin, vimentin, and transcription factor TWIST1. Further examination of the mechanism revealed that the nuclear factor κB (NF-κB) pathway is involved in this regulation of EMT-related biomarkers. Gambogic acid inhibited NF-κB p65 nuclear translocation and the phosphorylation of the inhibitor of NF-κB (IκBα) and IκBα kinase (IKKα). Gambogic acid also suppressed the EMT induced by TGFβ1 and tumor necrosis factor α by inhibiting the NF-κB pathway. Our data also indicate that gambogic acid inhibited the primary lesion and lung metastasis of orthotopic model of A549 cells *in vivo*. We propose that gambogic acid might be developed as a candidate drug with therapeutic potential for the treatment of cancer invasion and migration.

## INTRODUCTION

Cancer invasion and metastasis are critical events that change a locally growing tumor into a systemic disease [1]. Metastasis is responsible for up to 90% of cancer-related mortality [2]. The main steps in invasion include changes in cell–cell adhesion and the activation of cytoskeletal dynamics, followed by plastic tumor cell migration into the extracellular matrix (ECM) [1]. The epithelial-to-mesenchymal transition (EMT) is the primary invasive process, with a loss of cell–cell junctions and changes in the cell morphology [3]. During EMT, the intercellular adhesion of nonmotile, polarized, and collective epithelial cells is reduced and they are converted into motile, nonpolarized mesenchymal cells [4]. These cells acquire migratory and invasive properties and modulate the organization of their actin cytoskeletons. The molecular markers of the cells also change dramatically. The epithelial molecular markers E-cadherin and the cytokeratins are lost, whereas the mesenchymal molecular markers N-cadherin and vimentin are induced [5]. EMT plays key roles in embryonic development, but its importance in the progression of cancer is increasingly recognized. It is related not only to the invasiveness of cancer, but also to its metastatic dissemination, resistance to apoptosis, and acquisition of cancer stem-cell-like properties [6, 7].

Transforming growth factor β1 (TGFβ1) is the inducer of EMT most commonly used in *in vitro* studies [8]. The roles TGFβ1 and EMT in cancer progression have been investigated by many tumor biologists, but the mechanism of TGFβ1-induced EMT is not completely understood. TGFβ1-induced signaling can activate multiple distinct intracellular effector molecules, including the direct ligand-activated receptors of the SMAD transcription factors and the indirect NF-kB pathway [9]. TGFβ-activated kinase 1 (TAK-1) is a MAPKK kinase activated by TGFβ1 [10], which is a stimulus for the NF-kB pathway [11]. Once activated, IKK phosphorylates IkBa, and NF-kB is released. The NF-kB heterodimer p65 is then translocated to the nucleus and binds to its target promoter [12]. p65 can bind to the promoter of the E-cadherin transcriptional repressor ZEB-1/2, thus regulating EMT [13]. p65 can also regulate *TWIST1* transcription by binding directly to the *TWIST1* promoter [14]. TWIST1, a known key regulator of morphogenesis, can also induce EMT [15]. Therefore, the activated NF-kB pathway in EMT leads to the activation of the transcriptional regulator TWIST, which regulates the expression of E-cadherin. The loss of E-cadherin results in EMT and the disruption of cell–cell adhesion, which is considered to be the initiator of tumor cell migration and invasion [16].

Gambogic acid (GA) is a potential antitumor compound that is extracted from the resin of *Garciania hanburyi*. GA inhibits angiogenesis by suppressing the tyrosine phosphorylation of vascular endothelial growth factor receptor-2 [17] and inhibiting the PHD2–VHL–HIF1a pathway [18]. The molecular mechanism of its anti-invasive effect could be the inhibition of matrix metalloproteinase-2 (MMP-2) and MMP-9 in human breast carcinoma cells [19, 20]. GA also inhibits cell adhesion, but exerts an antimetastatic effect by suppressing integrin b1 and reducing the cholesterol content of cells [21].

In this study, we tested the anti-invasive and anti-migratory capacities of GA in TGFb1-induced A549 cells and clarified its effects on the EMT process. Because EMT is a crucial step in the migration and invasion of cancer cells, the effects of GA on EMT biomarkers and EMT-related transcription factors were also investigated.

## RESULTS

### GA inhibits TGFβ1-induced migration and invasion by A549 cells *in vitro*

**Figure 1:**
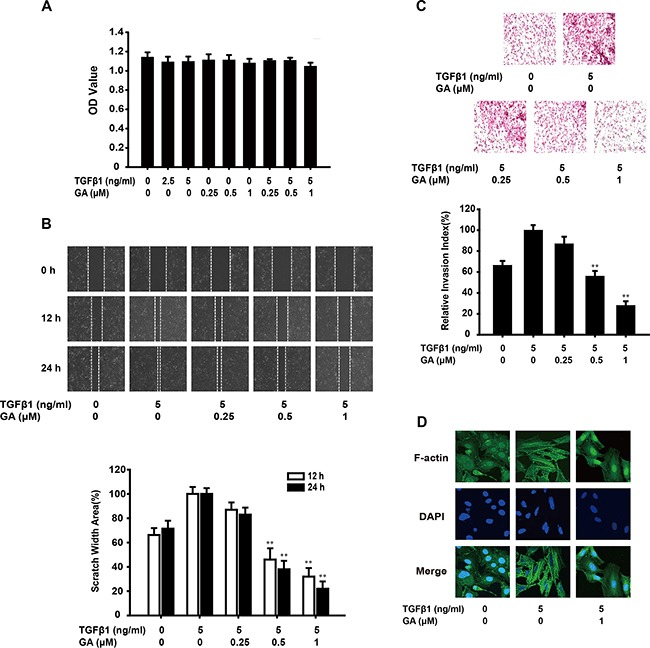
GA inhibits TGFβ1-induced migration and invasion of A549 cells *in vitro* **(A)** Cells were exposed to different concentrations of GA and TGFβ1 for 24 h in a 5% CO_2_ incubator at 37°C. An MTT assay then showed that GA and TGFβ1 had no effect on cell viability. **(B)** A monolayer of A549 cells was scraped with a pipette tip and then treated with different concentrations of GA and TGFβ1 for 12 or 24 h. The migrating cells were assessed with a microscope equipped with a camera. The results show that GA inhibits TGFβ1-induced cell migration (image magnification: 100×). **(C)** After A549 cells were treated with different concentrations of GA and TGFβ1 for 24 h, their invasive ability was evaluated with a Matrigel-based *in vitro* invasion assay. GA inhibited TGFβ1-induced cell invasion (image magnification: 200×). **(D)** A549 cells were treated with different concentrations of GA and TGFβ1 for 24 h, and a FITC–phalloidin staining assay was performed to observe the structure of F-actin with confocal microscopy (image magnification: 400×). Each experiment was performed at least three times. *p < 0.05 compared with the TGFβ1-treated group; **p < 0.01 compared with the TGFβ1-treated group.

An MTT assay showed that GA or TGFβ1 at various concentration had almost no effect on the growth of A549 cells. The combination of GA (0.25, 0.5, or 1 μM) and TGFβ1 (5 ng/ml) also had no cytotoxic effect (Figure 1A). These concentration ranges were used in all following experiments.

EMT imbues cancer cells with invasive and migratory properties. Therefore, we investigated the anti-invasive and anti-migratory effects of GA on TGFβ1-triggered EMT with Matrigel invasion and wound healing assays. GA inhibited the migration of TGFβ1-stimulated A549 cells across the wounded space in a concentration-dependent manner (16.7%, 61.5%, and 77.8%, respectively) (Figure 1B). Treatment with GA also reduced the invasiveness of A549 cells through Matrigel. The rates of inhibition were about 13.2%, 43.6%, and 71.8% (Figure 1C). A FITC–phalloidin staining assay was performed to observe the structure of F-actin. GA (1 μM) suppressed TGFβ1-induced changes in cell morphology and actin remodeling (Figure 1D).

These results indicate that GA inhibits TGFβ1-induced migration and invasion by A549 cells *in vitro*.

### GA regulates the mRNA expression of EMT-related biomarkers and transcription factor TWIST1 expression

**Figure 2:**
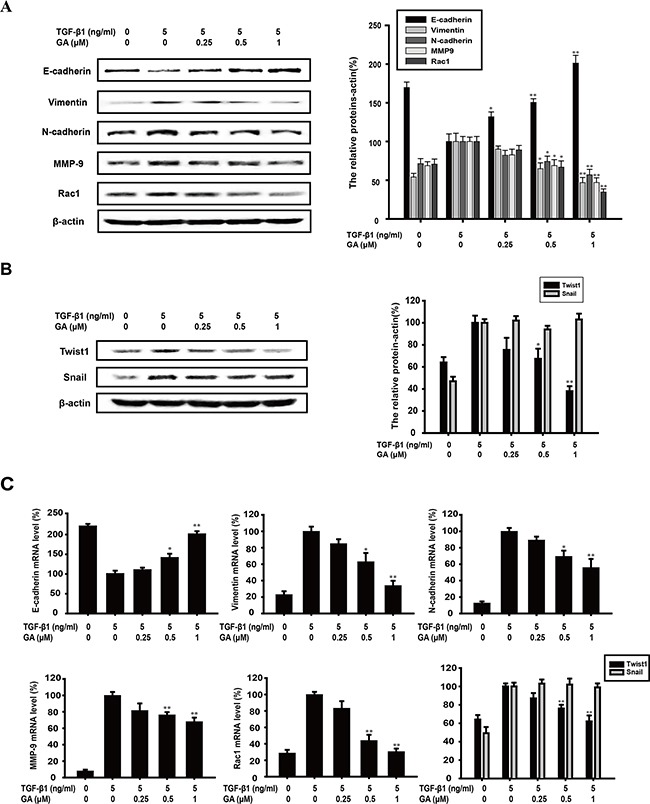
GA regulates the expression of EMT-related biomarkers and transcription factor TWIST1 A549 cells were treated with the indicated concentrations of GA and TGFβ1 for 24 h. **(A)** A549 cells were treated with the indicated concentrations of GA and TGFβ1 for 24 h. The expression of E-cadherin, vimentin, N-cadherin, MMP9, and RAC1 proteins in the cells was analyzed by western blotting using specific antibodies. An anti-β-actin antibody was used to check equivalent protein loading. **(B)** The expression of transcription factors TWIST1 and SNAIL was measured with western blotting. The values under each lane indicate the relative densities of the bands normalized to that of β-actin. **(C)** E-cadherin, vimentin, N-cadherin, MMP9, RAC1 and TWIST1 and SNAIL mRNAs were measured with real-time PCR. β-Actin was used as the internal control. The relative levels were calculated as the ratio of the relative biomarker mRNA to β-actin mRNA. Each experiment was performed at least three times. *p < 0.05 compared with the TGFβ1-treated group; **p < 0.01 compared with the TGFβ1-treated group.

E-Cadherin is an epithelial biomarker, whereas vimentin is a mesenchymal biomarker. The switch between E-cadherin and N-cadherin is also a crucial process during TGFβ1-induced EMT. As shown in Figure 2A, GA (0.25, 0.5, or 1 μM) increased E-cadherin expression (by 31.9%, 50.5%, or 101.1%, respectively) and reduced vimentin (by 9.9%, 35.1%, or 53.1%) and N-cadherin expression (by 17.9%, 25.8%, or 43.0%) in a concentration-dependent manner, compared with the TGFβ1-treated group. Cancer cells can also acquire an invasive capacity when EMT occurs, which upregulates the level of the invasion-related protein MMP-9 and the migration-related protein RAC1. Our experiments also revealed that GA suppressed the expression of MMP9 (by 17.2%, 31.1%, or 53.0%) and RAC1 (by 10.9%, 33.2%, or 65.4%) in a concentration-dependent manner (Figure 2A).

The mRNA levels of EMT biomarkers were determined with real-time PCR after A549 cells were treated with GA and TGFβ for 24 h. Our data showed that GA (0.25, 0.5, and 1 μM) increased E-cadherin mRNA expression (by 9.9%, 40.8%, and 100.8%, respectively) and reduced vimentin (by 14.7%, 37.2%, and 66.1%), N-cadherin (by 10.7%, 30.5%, and 44.1%), MMP-9 (by 18.4%, 23.7%, and 32.0%), and RAC1 mRNA expression (by 16.7%, 56.0%, and 69.5%) in a concentration-dependent manner compared with that of the TGFβ1-treated control group (Figure 2C).

The mRNA and protein levels of TWIST1 and SNAIL, which are transcriptional repressors of E-cadherin and EMT regulators, were investigated. Our results show that GA (0.25, 0.5, and 1 μM) suppressed TWIST1 protein (by 24.6%, 32.7%, and 62.0%) and mRNA expression (12.9%, 24.1%, and 37.5%) in a concentration-dependent manner (Figure 2B and Figure 2C), but had no effect on SNAIL protein or mRNA expression (Figure 2B and Figure 2C). Moreover, after transfection with TWIST1 plasmid, the protein level of TWIST1 was overexpressed and GA could also decrease TWIST1 expression (Supplementary Figure 1G).

In addition, TGFβ1-induced Panc-1 cells and TGFβ1+TNFa-induced SW480 cells were used to further prove the inhibitory effect of GA on EMT. GA could decrease the expression of vimentin and TWIST1 while increase E-cadherin expression in Panc-1 and SW480 cells, which further inhibited EMT-triggered migration and invasion (Supplementary Figure 1A-1F).

### GA inhibits the TGFβ1-induced activation of the NF-κB pathways in A549 cells

**Figure 3:**
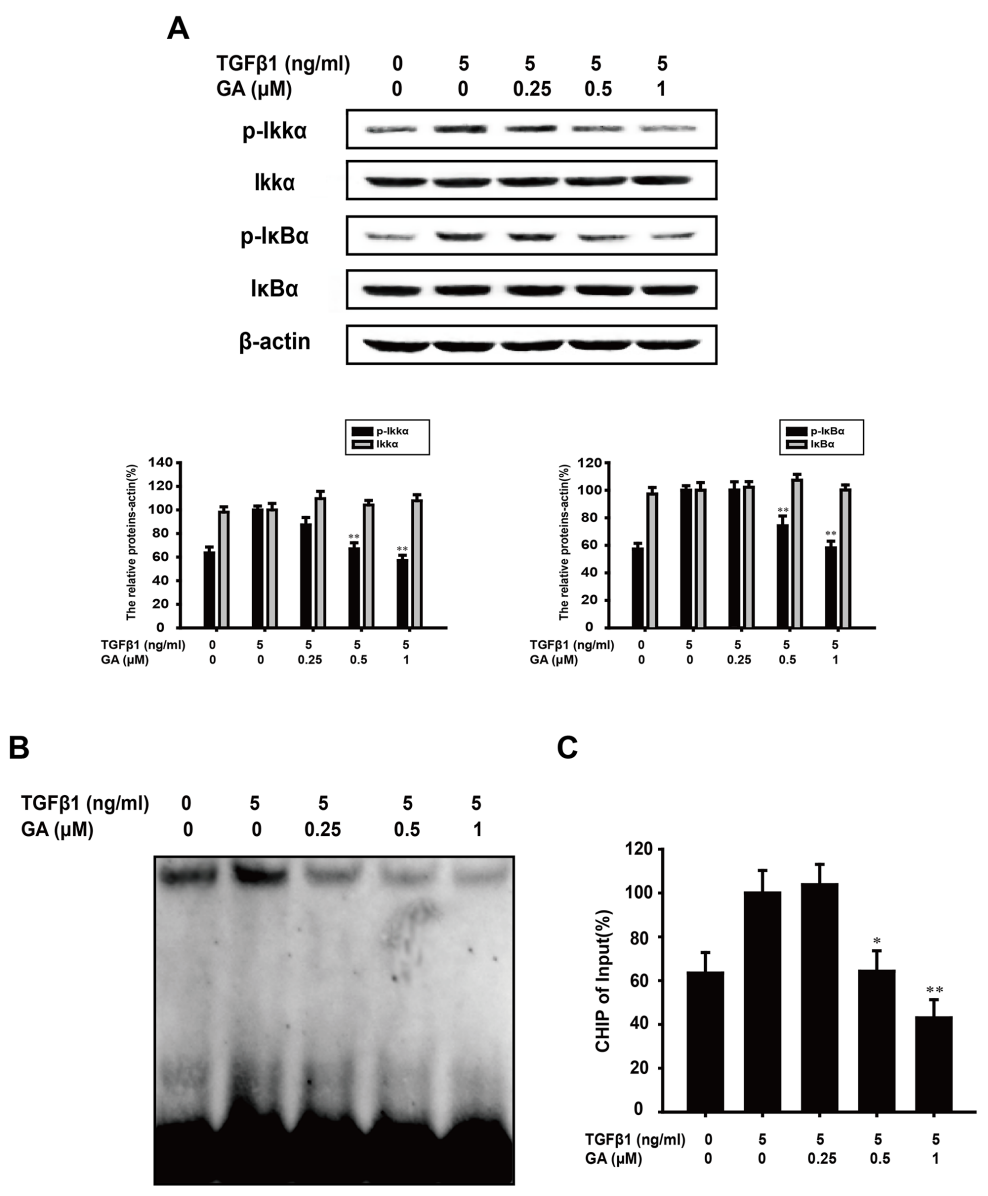
GA inhibits TGFβ1-induced activation of the NF-κB pathways in A549 cells A549 cells were treated with the indicated concentrations of GA and TGFβ1 for 24 h. **(A)** Western blotting analyses of p-IKKα, IKKα, p-IκBα, and IκBα were performed with whole-cell lysates and specific antibodies. Anti-β-actin antibody was used to confirm equivalent protein loading. **(B)** Nuclear extracts wereprepared and assayed for NF-κB activation with EMSA, using 8 μg of nuclear extract. **(C)** Immunoprecipitated DNA was extracted and dissolved and then DNA was measured by RT-PCR. The relative levels were calculated as the ratio of TWIST to β-actin mRNA expression, compared with the Input. Each experiment was performed at least three times. *p < 0.05 compared with the TGFβ1-treated group; **p < 0.01 compared with the TGFβ1-treated group.

TGFβ1 can activate NF-kB signaling via TAK-1, and NF-kB activation results from the phosphorylation, ubiquitination, and ultimately proteolytic degradation of IkBa. Continuous stimulation can induce TWIST1 expression, and then promote rearrangement of cytoskeleton and breakage of cell-cell junctions. In Figure 3A, compared with the TGFβ1-treated group, GA (0.25, 0.5, or 1 μM) inhibited IkBa phosphorylation (by 0.6%, 25.9%, or 41.9%, respectively) in a concentration-dependent manner. As phosphorylated IKKa is responsible for the phosphorylation of IkBa, we next tested the effect of GA on IKKa activation. We found that GA suppressed the TGFβ1-induced phosphorylation of IKKa (by 12.7%, 32.9%, or 42.8%) in a concentration-dependent manner (Figure 3A). Furthermore, GA could also inhibit the activation of NF-kB signaling in Panc-1 and SW480 cells (Supplementary Figure 1E-1F).

The effect of GA on TGFβ1-induced NF-kB DNA-binding activity was then evaluated with an EMSA assay. We found that GA suppressed the DNA-binding activity of TGFβ1-induced NF-kB in A549 cells (Figure 3B). The binding of NF-kB with downstream promoters, especially transcription factor TWIST, is crucial for inducing EMT. Thus, we tested the influence of GA on the binding ability of NF-kB with the TWIST promoter in A549 cells. The results of CHIP (Figure 3C) indicated that GA could inhibit the binding with TWSIT-promoter.

### GA inhibits TGFβ1+TNFα-induced EMT and invasion of A549 cells

**Figure 4:**
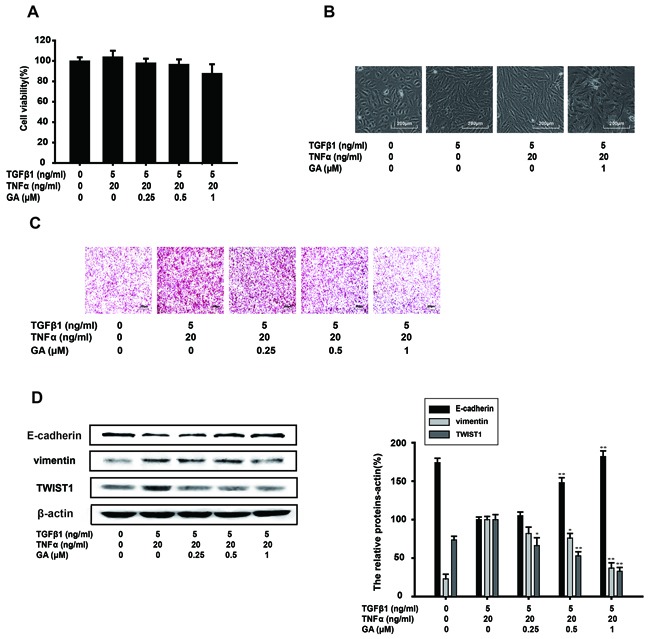
GA inhibits TGFβ1+TNFα-induced EMT and the invasion of A549 cells A549 cells were treated with the indicated concentrations of GA, TGFβ1, and TNFα for 24 h. **(A)** An MTT assay showed that GA, TGFβ1, and TNFα had no effect on cell viability. **(B)** Morphological observation of A549 cells in the presence of the indicated concentrations of GA, TGFβ1, and TNFα with microscopy (image magnification: 100×). **(C)** An *in vitro* cell invasion assay showed that GA inhibits TGFβ1+TNFα-induced cell invasion (image magnification: 200×). **(D)** The expression of E-cadherin, vimentin, and TWIST1 proteins in the cells was analyzed with western blotting using specific antibodies. Anti-β-actin antibody was used to confirm equivalent protein loading. Each experiment was performed at least three times. *p < 0.05 compared with the TGFβ1+TNFα-treated group; **p < 0.01 compared with the TGFβ1+TNFα-treated group.

NF-kB activation is induced by various inflammatory stimuli, including TNFa, which is also an inducer of EMT. To confirm the inhibitory effect of GA on EMT, TNFα and TGFβ1 (TGFβ1+TNFa) were together used to enhance the EMT process in A549 cells induced by TGFβ1 alone. The results of a cell viability assay showed that GA (0.25, 0.5, or 1 μM) had almost no effect on the growth of TGFβ1 (5 ng/ml)-induced and TNFα (20 ng/ml)-induced A549 cells (Figure 4A). The normal shape of the A549 cells was pebbled under a microscope. After treatment with TGFβ1, the A549 cells became spindle shaped. TGFβ1+TNFα conferred a needle-like shape on A549 cells. However, GA (1 μM) inhibited this shape change and the A549 cells became diamond shaped in the presence of TGFβ1+TNFα (Figure 4B). A Matrigel invasion assay was performed to investigate whether GA inhibits TGFβ1+TNFα-induced invasion by these cells. GA inhibited the invasion of TGFβ1+TNFα-induced A549 cells (Figure 4C).

EMT biomarkers were then tested with a western blot analysis. By blocking the cell response to TGFβ1+TNFα treatment, GA restored E-cadherin, vimentin and TWIST1 protein expression to basal levels in a concentration-dependent manner (Figure 4D).

### GA inhibits TGFβ1+TNFα-activated NF-κB signaling in A549 cells

**Figure 5:**
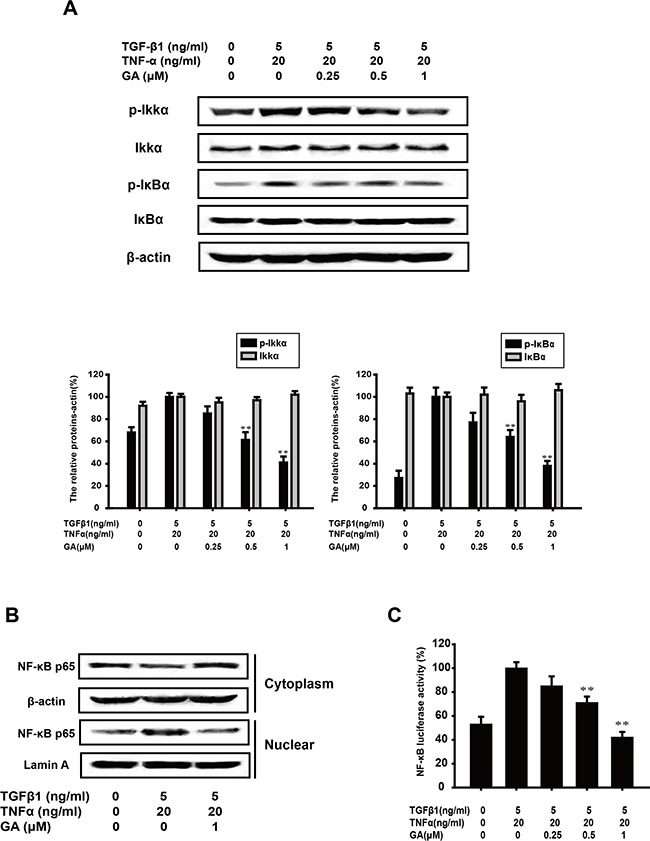
GA inhibits TGFβ1+TNFα-activated NF-κB signaling in A549 cells **(A)** A549 cells were treated with the indicated concentrations of GA, TGFβ1, and TNFα for 24 h. Western blotting analyses of p-IKKα, IKKα, p-IκBα, and IκBα were performed with whole-cell lysates and specific antibodies. Anti-β-actin antibody was used to confirm equivalent protein loading. (**B**) Cytosolic fractions and nuclear extracts were prepared from cells treated with the indicated concentrations of GA, TGFβ1, and TNFα for 24 h. Western blotting analysis of NF-κB p65 was performed to investigate the nuclear translocation of NF-κB p65. **(C)** A549 cells were transiently transfected with an NF-κB reporter gene plasmid for 5 h. After transfection, the cells were washed and treated with the indicated concentrations of GA, TGFβ1, and TNFα for 24 h. The luciferase activities were detected as described in the Materials and methods. Each experiment was performed at least three times. *p < 0.05 compared with the TGFβ1+TNFα-treated group; **p < 0.01 compared with the TGFβ1+TNFα-treated group.

TGFβ1-induced EMT is accentuated by TNFα via NF-κB signaling [27]. The combination of TGFβ1 and TNFα is usually used to further drive EMT. Therefore, we examined whether GA inhibits the key protein of the NF-κB pathway activated by TGFβ1+TNFα. GA (0.25, 0.5, or 1 μM) suppressed the TGFβ1+TNFα-induced phosphorylation of IKKα (by 14.5%, 47.3%, or 58.8%, respectively) and IκBα (by 22.8%, 36.2%, or 62.1%) in a concentration-dependent manner (Figure 5A).

The translocation of p65, the functionally active subunit of NF-κB, was also examined with a western blot assay. As shown in Figure 5B, the increase in nuclear p65 expression induced by TNFα+TGFβ1 was inhibited by GA (1 μM), whereas the cytoplasmic p65 expression increased.

NF-κB activation was then tested with a luciferase reporter construct. The results showed that GA inhibited p65 luciferase activity (by 14.2%, 28.8%, or 57.7%) stimulated with TGFβ1+TNFα, in a concentration-dependent manner (Figure 5C).

### Inhibition of TGFβ1-induced EMT by GA does not require MAPK pathway

**Figure 6:**
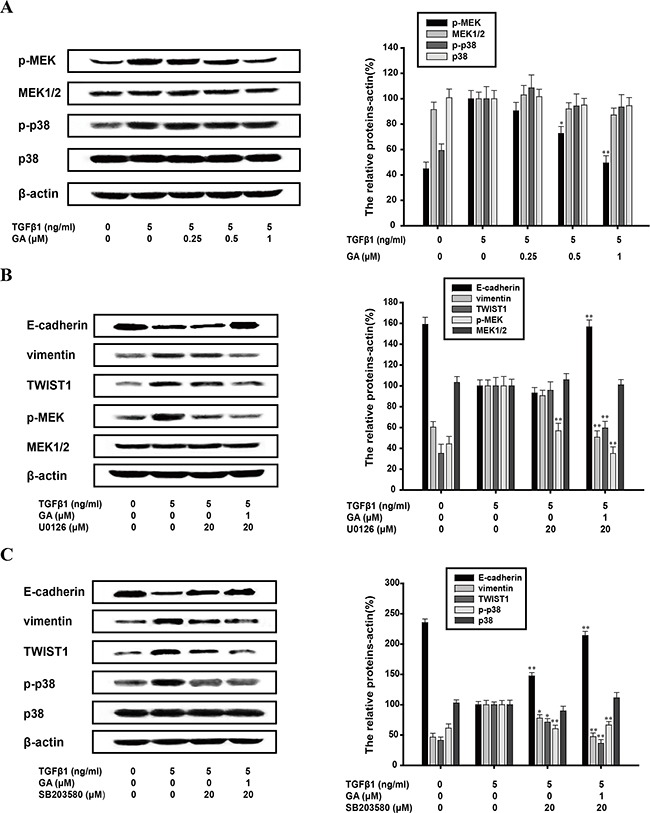
Inhibition of TGFβ1-induced EMT by GA does not require MAPK pathway A549 cells were treated with the indicated concentrations of GA, TGFβ1, U0126 and SB203580 for 24 h. **(A)** Western blotting analyses of p-MEK, MEK1/2, p-p38 and p38 were performed with whole-cell lysates and specific antibodies. **(B)** Western blotting analyses of E-cadherin, vimentin, TWIST1, p-MEK and MEK1/2 were performed with whole-cell lysates and specific antibodies. **(C)** Western blotting analyses of E-cadherin, vimentin, TWIST1, p-p38 and p38 were performed with whole-cell lysates and specific antibodies. Anti-β-actin antibody was used to confirm equivalent protein loading. Each experiment was performed at least three times. *p < 0.05 compared with the TGFβ1-treated group; **p < 0.01 compared with the TGFβ1+TNFα-treated group.

The MAPK pathway is also important in the EMT process, of which MEK/ERK and p38 MAPK pathways are both hotspots. Activation of ERK and p38 MAPK pathways are required for TGFβ1-induced EMT [28, 29]. GA (0.25, 0.5, or 1 μM) exerted an inhibitory effect on the phosphorylation of MEK1/2 and the inhibitory rate of phospho/total forms of MEK1/2 was 9.6%, 27.4%, and 50.5%, respectively. However, it had no effect on the phosphorylation of p38 (Figure 6A). Therefore, we investigated the effects of GA on EMT protein markers when p38 MAPK or the MEK1/2 pathway was inhibited. The EMT biomarkers were investigated with a western blot analysis. U0126 (20 μM), an MEK1/2 inhibitor, did not reverse the process of EMT, whereas the addition of GA (1 μM) increased E-cadherin expression (by 56.7%) and reduced vimentin (by 49.7%) and TWIST1 expression (41.5%) (Figure 6B). SB203580 (20 μM), a p38 MAPK inhibitor (the inhibitory rate of phospho/total forms of p38 was 38.2%), increased E-cadherin expression (by 47.4%) and slightly reduced vimentin (by 23.1%) and TWIST1 (by 29.8%) expression, but this effect was enhanced by GA (Figure 6C). GA and SB203580 increased E-cadherin expression (by 114.1%) and reduced vimentin (by 53.6%) and TWIST1 (by 64.7%) expression with TGFβ1 stimulation (Figure 6C).

### Inhibition of TGFβ1-induced EMT by GA does not require SMAD pathway or the transferrin receptor (TfR)

**Figure 7:**
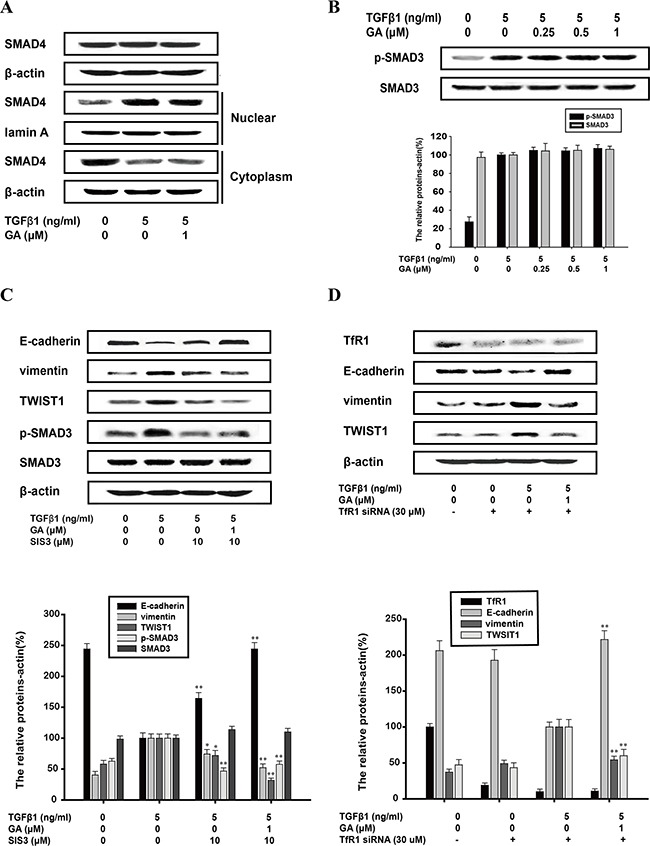
Inhibition of TGFβ1-induced EMT by GA does not require SMAD pathway or the transferrin receptor A549 cells were treated with the indicated concentrations of GA, TGFβ1 and SIS3 for 24 h. **(A)** Cytosolic fractions and nuclear extracts were prepared. Western blotting analysis of total SMAD4 and the nuclear translocation of SMAD4 were performed with specific antibodies. **(B)** Western blotting analyses of p-SMAD3 and SMAD3 were performed with whole-cell lysates and specific antibodies. **(C)** Western blotting analyses of E-cadherin, vimentin, TWIST1, p-SMAD3 and SMAD3 were performed with whole-cell lysates and specific antibodies. **(D)** A549 cells were transfected with transferrin receptor siRNA and scrambled siRNA, and TfR mRNA level is used to measure transfection efficiency. When cells are transfected at efficiencies of 50–60%, western blotting analyses of transferrin receptor, E-cadherin, vimentin, TWIST1 were performed with whole-cell lysates and specific antibodies. Anti-β-actin antibody was used to confirm equivalent protein loading. Each experiment was performed at least three times. *p < 0.05 compared with the TGFβ1+TNFα-treated group; **p < 0.01 compared with the TGFβ1-treated group.

Ligand-activated SMAD signaling is the direct target of TGFβ1. Our western blotting analysis showed that GA had little effect on the nuclear translocation of SMAD4, or on the phosphorylation or total protein of SMAD3 (Figure 7A and 7B). This indicates that GA does not inhibit the EMT process directly *via* TGFβ1–SMAD signaling. SIS3 (10 μM), an inhibitor of the SMAD pathway (the inhibitory rate of phospho/total forms of p38 was 45.8%), increased E-cadherin expression (by 64.2%) and slightly reduced vimentin (by 26.5%) and TWIST1 (by 28.9%) expression, and these effects were enhanced by GA (Figure 7C). GA and SIS3 increased E-cadherin expression (by 144.2%) and reduced vimentin (by 48.7%) and TWIST1 (by 69.4%) expression with TGFβ-1 stimulation (Figure 7C).

Gambogic acid is reported as a ligand for TfR in triggering apoptosis. We used TfR small interfering RNA (siRNA) to determine whether TfR participates in the inhibition of EMT by GA. Expression of these three marker proteins was unaffected whether cells were transfected with transferrin receptor siRNA or scrambled siRNA. TGFβ1-mediated reduction in E-cadherin expression and increase in vimentin and TWIST1 protein levels was refractory to the silencing of transferrin receptor, so GA-mediated reversal of TGFβ1 signaling still occurs in TfR siRNA-transfected cells (Figure 7D)

### GA suppresses lung growth and metastases of A549 cells *in vivo*

**Figure 8:**
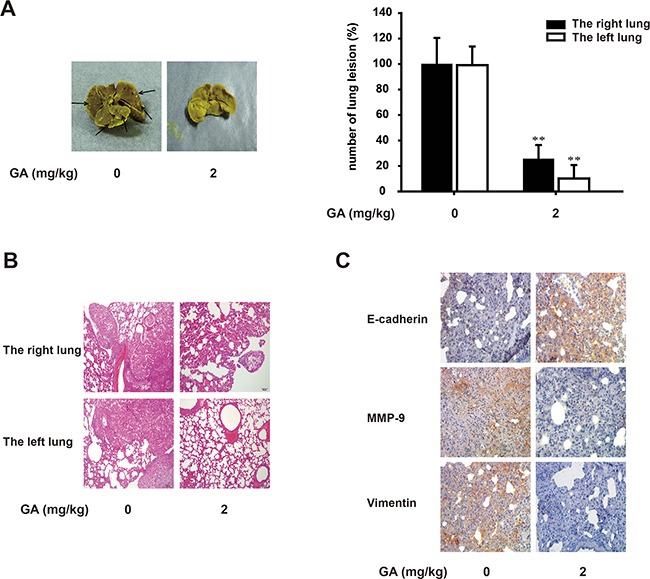
GA suppresses lung growth and metastasis of A549 cells *in vivo* Murine lung orthotopic model of A549 cells was conducted to determine the effect of GA *in vivo*. **(A)** Macroscopic lung lesions were detected. After the lungs were fixed in Bouin's solution, the numbers of lung metastatic nodules on the lung surfaces were quantified. **(B)** H&E stained bilateral lungs of mice from GA-treated and control group to confirm the malignance of nodules were and the presence of micrometastases. Each bar represents 100 μm. **(C)** Immunohistochemical detection of E-cadherin, MMP-9 and vimentin protein levels in the right lung (100×). Each experiment was performed at least three times. *p < 0.05 compared with the control; **p < 0.01 compared with the control.

The antitumor and antimigratory effects of GA were further assessed with A549 orthotopic model *in vivo*. Intraperitoneal injection of GA is adopted for good absorption when long-term administration. The dosage of GA *in vivo* is determined by LD50 (half lethal dose) in nude mice and it is less than 1/6 of LD50. Afterwards, lung lesions were macroscopically observed with a camera (Figure 8A). We found from the HE staining that the right lung lesions in the GA (2 mg/kg) treatment group were fewer and smaller than those in the control group (Figure 8B). The expression of E-cadherin, MMP-9 and vimentin (Figure 8C) in right lung was stained by immunohistochemistry assay from control mice, whereas GA-treated group reversed the expression of these EMT biomarkers, which is consistent with the results of *in-vitro* study. On the other hand, GA also significantly reduced the metastatic nodules compared with those in the control group by 89.9% (Figure 8A). Meanwhile, HE staining showed that tumors of left lungs were reduced pathologically with the treatment of GA.

## DISCUSSION

Cancer metastasis is the primary cause of cancer-related death. The acquisition of invasive and migratory behavior by cells, which initiates the metastatic process [30], is mediated by the tumor microenvironment [31]. Various host cell types are found in this microenvironment, including endothelial cells, fibroblasts, and leukocytes [32]. The release of cytokines and proteases by these cells makes the microenvironment more reactive, promoting the growth of tumors and the escape of cancer cells from the primary tumor. TGFβ1, one of the important cytokines, acts as a suppressor early in tumor progression, whereas it acts as a tumor promoter in later stages [33]. The effect of TGFβ1 as a tumor promoter reflects its ability to induce EMT, which contributes to the invasion and migration of cancer cells. Tumor EMT is a process in which cancer cells lose their cell–cell junctions and acquire invasive and migratory properties. The primary features of EMT include the downregulation of epithelial cell markers, the upregulation of mesenchymal cell markers and the reorganization of the cytoskeleton. Although EMT is known to be involved in cancer invasion, it is also related to other events in tumor progression. Therefore, EMT is considered a potential target of therapeutic interventions for cancer. Recent studies have shown that chemical compounds from natural products, such as grape seed proanthocyanidin and resveratrol, may offer a potential resource for the interruption of EMT. In this study, we have demonstrated that GA inhibits the migration, invasion, and cytoskeletal remodeling of TGFβ1-induced A549 cells *in vitro*, so GA exerted general anti-migratory and anti-invasive activities during EMT.

GA suppresses the expression of TWIST1 mRNA and protein. TWIST1, a key transcription factor in EMT, contributes to EMT by regulating E-cadherin-mediated cell–cell adhesion and the activation of mesenchymal markers. Consistent with the functions of TWIST1, our results demonstrate that GA increases the epithelial cell marker E-cadherin expression, whereas it inhibits the expression of the mesenchymal cell markers vimentin and N-cadherin during TGFβ1-induced EMT. This regulation of EMT biomarkers by GA further indicates that it could inhibit the EMT process in TGFβ1-induced A549 cells. Furthermore, GA functions at the level of *TWIST1* transcription. The TGFβ1-induced expression of TWIST1 resulted in EMT and EMT initiated the invasion and migration of A549 cells. Twist promotes the formation of pseudopodia, and specialized membrane protrusions for extracellular matrix degradation [34]. MMP-9 plays a crucial role in ECM degradation and Rac1 plays key roles in cytoskeletal reorganization and pseudopodia formation. Following, we sought to clarify the mechanism by which GA inhibits TGFβ1-induced EMT.

p65 is a multifunctional transcription factor that regulates the expression of multiple target genes during tumor invasion and migration [35]. NF-κB is essential for both the induction and maintenance of EMT [36]. Li also reported that p65 binds directly to the *TWIST1* promoter to regulate its expression and that the *TWIST1* promoter sequence from −970 to +1 contains four p65-binding sites [14]. Therefore, we investigated whether GA inhibits the EMT process in TGFβ1-induced A549 cells by suppressing NF-κB activation. Our results showed that GA inhibited the phosphorylation of IKKα and IκBα induced by TGFb1, which suggested the inhibition of NF-κB signaling. p65 is one of the dimeric proteins that make up the transcriptional regulator NF-κB. In our EMSA experiment, GA inhibited the DNA-binding activity of p65. A chromatin immunoprecipitation assay is needed to further characterize the role of p65 in the regulation of *TWIST1* mRNA expression.

To confirm the inhibition of EMT by GA through the NF-κB pathway, TNFα was used as an NF-κB activator to accelerate TGFβ1-induced EMT in A549 cells. The shape of A549 cells is more needle-like when treated with TGFβ1+TNFα than when treated with TGFβ1 alone. GA had a slight inhibitory effect on the growth activity of A549 cells in the presence of TNFα+TGFβ1, but actively suppressed EMT-related biomarkers, thus blocking the invasion and migration of the A549 cells. Expression of the epithelial cell marker E-cadherin increased and that of the mesenchymal cell marker vimentin and the transcription factor TWIST1 decreased. The phosphorylation of IKKα and IκBα by TGFβ1+TNFα was inhibited by GA in a concentration-dependent manner. GA also inhibited the activation and nuclear localization of NF-κB p65, which indicates that GA suppresses p65 binding to the *TWIST1* promoter.

TGFb activates other non-SMAD signaling pathways such as MAPK pathways [37]. The MAPK pathway is also a crucial mediator of EMT and we showed that GA had an inhibitory effect on the MEK, but GA did not affect p38MAPK pathway. Although GA inhibited the phosphorylation of MEK1/2 after stimulation with TGFβ1, an inhibitor of the MEK1/2 pathway did not affect TGFβ1-induced EMT. This result resembles the inhibition of MEK/ERK by U0126 in HK-2 cells [38]. The addition of GA inhibits EMT process. Therefore, MEK/ERK pathway was not involved as the main mechanism of the inhibition of EMT by GA. A blockage of p38MAPK pathway could inhibit EMT and GA accelerated the inhibition of EMT by SB203580, for the reason that the effect of p38 inhibition on E-cadherin expression is modulated by the TAK1-NF-κB pathway. Ligand-activated SMAD signaling is the direct target of TGFβ1, but GA had little effect on SMAD signaling. SIS3 and GA treatment could further inhibit EMT. GA could inhibit EMT through another pathway rather than SMAD pathway, which indicated the specificity of the NF-κB pathway in the inhibition of EMT by GA [39].

TfR is reported to be significantly overexpressed in different types of cancers and GA is thought to be a ligand of TfR in cell apoptosis [40]. We found that the inhibition of EMT by GA is not independent of TfR expression and that silencing the TfR gene did not influence EMT markers. Therefore, TfR does not participate in this effect of GA. We attributed this to the concentration of GA used in this study, which did not cause apoptosis in A549 cells.

Previously, we found that GA exerts an inhibitory effect on the migration of cancer cells *in vivo* in a spontaneous experimental A549 metastasis model [41]. In this study, we use orthotopic model of A549 cells to investigate the inhibitory effect on tumor growth and EMT. Our results show that GA suppresses lung growth and metastasis of A549 cells and EMT biomarkers were improved, confirming the effects of GA on the motility of A549 cells *in vivo*.

In summary, suppressing the activity of E-cadherin repressors is an obvious strategy to avert cancer progression. Cancer therapeutic drugs that inhibit the EMT process have potential utility. GA inhibits NF-κB signaling, thus blocking the TGFβ1-induced EMT process by suppressing TWIST1 expression. As a consequence, GA can suppress the invasion and migration of tumor cells. Therefore, GA may be a potential therapeutic agent for the treatment of tumor invasion by inhibiting EMT.

## MATERIALS AND METHODS

### Materials

GA (99% purity or higher) had been isolated from the resin of *Garciania hanburyi* and purified according to the established methods [22]. The GA was prepared as described previously [19]. Human recombinant TGFb1 and tumor necrosis factor α (TNFα) were from ProSpec-Tany TechnoGene Ltd. (ProSpec, Israel). U0126 and SB203580 were from Beyotime (Shanghai, China). SIS3, and primary antibodies directed against IκBα, β-actin, MMP-9, RAC1, TWIST1, p38, p-p38 and NF-κB (p65) were from Santa Cruz Biotechnology (Santa Cruz, CA), antibodies directed against phosphorylated IκBα (p-IκBα; Ser32), MEK, p-MEK, IKKα, and p-IKKα/β (Ser176/180) were from Cell Signaling Technology (Danvers, MA), antibodies directed against transferritin receptor, SMAD4, lamin A, SNAIL, SMAD3, p-SMAD3(T179), E-cadherin, N-cadherin, TWIST1 and vimentin were from Bioworld (Bioworld, MN). IRDye®800-conjugated secondary antibodies were from Rockland Inc. (Philadelphia, PA).

### Animals

Five- to six-week-old male BALB/c nude mice (Slaccas Shanghai Laboratory Animal Co., Ltd., Shanghai, China) were used for the orthotopic model of A549 cells. The animals were maintained in a pathogen-free environment (23 ± 2°C, 55 ± 5% humidity) on a 12 h light/12 h dark cycle with food and water supplied *ad libitum* throughout the experimental period.

### Cell culture

Human alveolar adenocarcinoma A549 cells were originally obtained from the Cell Bank of the Shanghai Institute of Cell Biology. The A549 cells were cultured in F-12 medium (Gibco, Grand Island, NY) containing 10% fetal bovine serum (Gibco), 100 U/ml penicillin, and 100 mg/L streptomycin, in a stable environment with 5% CO_2_ at 37°C.

### Cell viability assay

Cells were plated at a density of 10^4^ cell/well in 96-well plates in F-12 medium with 10% fetal bovine serum. After overnight culture, the cells were exposed to different concentrations of TGFβ1 (0, 2.5, or 5 ng/ml) and GA (0, 0.25, 0.5, or 1 μM) for 24 h in a 5% CO_2_ incubator at 37°C. Then, MTT was added to the medium and the cells were incubated at 37°C for 4 h. The supernatant was removed and dimethyl sulfoxide was used to dissolve the precipitate. The absorbance was measured spectrophotometrically at 570 nm.

### Wound healing assay

A549 cells were seeded in a six-well plate and allowed to attach overnight, with growth to 80% confluence. The cell monolayers were then wounded with white pipette tips and washed twice with phosphate-buffered saline. The cells were then incubated in F-12 medium with TGFβ1 (0 or 5 ng/ml) and GA (0, 0.25, 0.5, or 1 μM) for up to 24 h. The number of migrated cells was determined under an inverted microscopy at 12 h and 24 h. Five randomly chosen fields were analyzed in each well. The percentage inhibition was expressed relative to TGFβ1-treated wells.

### Cell invasion assay

The Transwell chambers (Corning Costar, Cambridge, MA) were first loaded with 0.1 ml of Matrigel (Becton Dickinson, Bedford, MA) at 37°C for 1 h. After TGFβ1 (0 or 5 ng/ml) and GA (0, 0.25, 0.5, or 1 μM) were added to the A549 cells for 24 h, the cells were collected in F-12 medium at a final concentration of 2 × 10^5^ cell/ml. The cell suspensions were then placed in the upper compartment, and medium containing 10% fetal bovine serum was added to the lower compartment. Following incubation for 24 h, the invasive cells on the lower surface were stained with hematoxylin and eosin, and then quantified by manual counting and five randomly chosen fields were analyzed for each group.

### Immunofluorescence

A549 cells were pretreated with GA (0 or 1 μM) and TGFβ1 (0 or 5 ng/ml) for 24 h and then harvested. FITC–phalloidin was used to probe the samples for 1 h to analyze actin remodeling. After the cells were washed, they were stained with DAPI for 20 min. The samples were observed and images captured with confocal microscopy (Olympus Corp., Tokyo, Japan).

### Western blotting

A549 cells were harvested after TGFβ1 (0 or 5 ng/ml) and GA (0, 0.25, 0.5, or 1 μM) had been added to the cells for 24 h. Western blotting was performed as previously described [23]. The membrane was blocked with 10% BSA in PBS at 37°C for 1.5 h and incubated overnight at 4°C with the indicated antibodies, and then with IRDye®800-conjugated secondary antibody for 1 h at 37°C. The samples were visualized with the Odyssey Infrared Imaging System (LI-COR Inc., Lincoln, NE).

### Real-time PCR analysis

A549 cells were treated with TGFβ1 (0 or 5 ng/ml) and GA (0, 0.25, 0.5, or 1 μM) for 24 h. The mRNA levels of E-cadherin, N-cadherin, vimentin, MMP-9, RAC1, and TWIST1 were then determined with a method described previously [23]. The primer sets used for the PCR amplifications were as follows: E-cadherin (forward, 5’-CCACCAAAGTCACGCTGAAT-3’, reverse, 5’-GGAGTTGGGAAATGTGAGC-3’), SNAIL (forward, 5’-GCCTTCAACTGCAAATACTGC-3’, reverse, 5’-CTTCTTGACATCTGAGTGGGTC-3’), TWIST1 (forward, 5’-TGTCCGCGTCCCACTAGC-3’, reverse, 5’-TGTCCATTTTCTCCTTCTCTG-3’), N-cadherin (forward, 5’-AGCCTGGAACATATGTGATGA-3’, reverse, 5’-CCATAAAACGTCATGGCAGTAA-3’), MMP9 (forward: 5’-GCAGAGGAATACCTGTACC GC-3’, reverse, 5’-AGGTTTGGAATCTGCCCAGGT-3’), vimentin (forward, 5’-ATGAAGGTGCTGCAAAAC-3’, reverse, 5’-GTGACTGCACCTGTCTCCGGTA-3’), β-actin (forward, 5’-CTGTCCCTGTATGCCTCTG-3’, reverse, 5’-ATGTCACGCACGATTTCC-3’).

### Preparation of cytosolic and nuclear extracts

A549 cells were treated with TGFβ1 (0 or 5 ng/ml) and GA (0 or 1 μM) for 24 h. Nuclear and cytosolic protein extracts were prepared according to the modified method described previously [24].

### Electrophoretic mobility shift assays (EMSAs)

A549 cells were treated with TGFβ1 (0 or 5 ng/ml) and GA (0, 0.25, 0.5, or 1 μM) for 24 h. Nuclear extract preparation and EMSAs were performed as described previously [24]. Oligonucleotides containing an NF-κB-binding site were used in the EMSAs according to the manufacturer's instructions (Beyotime Institute of Biotechnology, Haimen, China). The oligonucleotides were labeled with biotin using standard protocols. The sequences of the oligonucleotides were as follows: 5’-AGTTGAGGGGACTTTCCCAGGC-3’ and 3’-TCAACTCCCCTGAAAGGGGTCCG-5’.

### Chromatin immunoprecipitation experiments

A549 cells were exposed to treated with TGFb1 (0 or 5 ng/ml) and GA (0, 0.25, 0.5, or 1 μM) for 24 h. Cells were cross-linked with formaldehyde, quenched with glycine, sonicated on ice, incubated with anti-TWIST or pre-immune IgG with rotation at 4°C overnight and then incubated with protein A+G agarose for 6 h. Then, immune complexes were capturedbby protein A+G agarose and eluted with elution buffer (1% SDS, 0.1mol/l NaHCO3) at 37°C for 30min. Cross-linking was reversed at 65°C for 4 h in a high-salt buffer. Extracted and dissolved immunoprecipitated DNA was quantified by RT-PCR. An equal volume of non-precipitated (input) genomic DNA was used to correct for the differences in PCR amplification efficiencies and amounts of DNA. Real-time PCR analyses were carried out as described above. The following promoter was used in this experiment: TWIST promoter (forward, 5’-CCG GGT ACC CTT TCA AGG TCA CAA TGC GGA GCC -3’, reverse, 5’-ATA CTC GAG TGG GCG AGA GCT GCA GAC TTG G -3’).

### NF-κB transient transfection and luciferase assay

The transient transfection assay of NF-κB was performed as described previously [24]. After transfection for 5 h, the A549 cells were washed with PBS and treated with TGFb1 (0 or 5 ng/ml) and GA (0, 0.25, 0.5, or 1 μM) for 24 h. A luciferase assay was used to measure as described previously [24].

### Small interfering RNA transfection

For transfection, A549 cells were seeded in 6-well plates at approximately 50–70% confluence. Either transferrin receptor siRNA duplexes (30 pmol/μl) or scrambled siRNA was introduced into the cells using siPORT NeoFX Transfection Agent (AbmionInc., Austin, TX) according to the manufacturer's recommendations. Transferrin receptor siRNA and scrambled siRNA were obtained from Santa Cruz Biotechnology (Santa Cruz, CA). Then, the cells were exposed to F-12 medium with TGFb1 (0 or 5 ng/ml) and GA (0 or 1 μM) for 24 h and harvested for further experiments.

### Orthotopic model of A549 cells

Orthotopic injections were performed following the previous study with minor modifications [25]. A549 cells (5 × 10^6^) in 50 μl matrigel were quickly injected into the thorax. Animal was observed for 30 min until fully recovery. Two weeks later, the mice were randomly divided into two groups (ten/group) and intraperitoneally injected every other day for 30 days with GA (2 mg/kg) or saline. The animals were then killed. The lungs were removed and fixed in Bouin's solution for 4 h, and the number of lesions was determined macroscopically. Sections of the lungs were stained with hematoxylin and eosin to confirm that the nodules were malignant and to monitor the presence of micrometastases.

### Immunohistochemistry

The expression of E-cadherin, vimentin and MMP-9 in right lung of nude mice model was assessed to the method described previously [26], using a goat-anti-rabbit antibody and an Ultra-Sensitive TMSAP kit. All reagents used in the experiments were supplied by Maixin-Bio Co., Fuzhou, China.

### Statistical analysis

The data shown were obtained from at least three independent experiments and all results are presented as means ± SEM. Differences between groups were assessed with one-way analysis of variance and Dunnett's *post hoc* test. Comparisons were made relative to the TGFβ1- or TGFβ1+TNFα-treated group *in vitro* and the saline-treated group *in vivo*. The significance of differences is indicated at *p < 0.05 and **p < 0.01.

## SUPPLEMENTARY FIGURE



## Abbreviations

EMT: epithelial-to-mesenchymal transition
TGFβ1: transforming growth factor β1
NF-κB: nuclear factor κB
ECM: extracellular matrix
TAK-1: TGFβ-activated kinase 1
GA: Gambogic acid
MMP: matrix metalloproteinase
TNFα: tumor necrosis factor α
TfR: transferrin receptor
siRNA: interfering RNA

## References

[1] Friedl P, Alexander S (2011). Cancer invasion and the microenvironment: plasticity and reciprocity. Cell.

[2] Chaffer CL, Weinberg RA (2011). A perspective on cancer cell metastasis. Science.

[3] Wirtz D, Konstantopoulos K, Searson PC (2011). The physics of cancer: the role of physical interactions and mechanical forces in metastasis. Nat Rev Cancer.

[4] Yilmaz M, Christofori G (2009). EMT, the cytoskeleton, and cancer cell invasion. Cancer metastasis reviews.

[5] Thiery JP, Sleeman JP (2006). Complex networks orchestrate epithelial-mesenchymal transitions. Nat Rev Mol Cell Biol.

[6] Thiery JP, Acloque H, Huang RY, Nieto MA (2009). Epithelial-mesenchymal transitions in development and disease. Cell.

[7] Bedi U, Mishra VK, Wasilewski D, Scheel C, Johnsen SA (2014). Epigenetic plasticity: a central regulator of epithelial-to-mesenchymal transition in cancer. Oncotarget.

[8] Zavadil J, Bottinger EP (2005). TGF-beta and epithelial-to-mesenchymal transitions. Oncogene.

[9] Polyak K, Weinberg RA (2009). Transitions between epithelial and mesenchymal states: acquisition of malignant and stem cell traits. Nat Rev Cancer.

[10] Yamaguchi K, Shirakabe K, Shibuya H, Irie K, Oishi I, Ueno N, Taniguchi T, Nishida E, Matsumoto K (1995). Identification of a member of the MAPKKK family as a potential mediator of TGF-beta signal transduction. Science.

[11] Ninomiya-Tsuji J, Kishimoto K, Hiyama A, Inoue J, Cao Z, Matsumoto K (1999). The kinase TAK1 can activate the NIK-I kappaB as well as the MAP kinase cascade in the IL-1 signalling pathway. Nature.

[12] Karin M, Cao Y, Greten FR, Li ZW (2002). NF-kappaB in cancer: from innocent bystander to major culprit. Nat Rev Cancer.

[13] Chua HL, Bhat-Nakshatri P, Clare SE, Morimiya A, Badve S, Nakshatri H (2007). NF-kappaB represses E-cadherin expression and enhances epithelial to mesenchymal transition of mammary epithelial cells: potential involvement of ZEB-1 and ZEB-2. Oncogene.

[14] Li CW, Xia W, Huo L, Lim SO, Wu Y, Hsu JL, Chao CH, Yamaguchi H, Yang NK, Ding Q, Wang Y, Lai YJ, LaBaff AM, Wu TJ, Lin BR, Yang MH (2012). Epithelial-mesenchymal transition induced by TNF-alpha requires NF-kappaB-mediated transcriptional upregulation of TWIST1. Cancer research.

[15] Yang J, Mani SA, Donaher JL, Ramaswamy S, Itzykson RA, Come C, Savagner P, Gitelman I, Richardson A, Weinberg RA (2004). Twist, a master regulator of morphogenesis, plays an essential role in tumor metastasis. Cell.

[16] Christofori G (2006). New signals from the invasive front. Nature.

[17] Lu N, Yang Y, You QD, Ling Y, Gao Y, Gu HY, Zhao L, Wang XT, Guo QL (2007). Gambogic acid inhibits angiogenesis through suppressing vascular endothelial growth factor-induced tyrosine phosphorylation of KDR/Flk-1. Cancer Lett.

[18] Lu N, Hui H, Yang H, Zhao K, Chen Y, You QD, Guo QL (2013). Gambogic acid inhibits angiogenesis through inhibiting PHD2-VHL-HIF-1alpha pathway. Eur J Pharm Sci.

[19] Lu N, Gao Y, Ling Y, Chen Y, Yang Y, Gu HY, Qi Q, Liu W, Wang XT, You QD, Guo QL (2008). Wogonin suppresses tumor growth *in vivo* and VEGF-induced angiogenesis through inhibiting tyrosine phosphorylation of VEGFR2. Life Sci.

[20] Zhang HW, Yang Y, Zhang K, Qiang L, Yang L, Hu Y, Wang XT, You QD, Guo QL (2008). Wogonin induced differentiation and G1 phase arrest of human U-937 leukemia cells via PKCdelta phosphorylation. Eur J Pharmacol.

[21] Li C, Lu N, Qi Q, Li F, Ling Y, Chen Y, Qin Y, Li Z, Zhang H, You Q, Guo Q (2011). Gambogic acid inhibits tumor cell adhesion by suppressing integrin beta1 and membrane lipid rafts-associated integrin signaling pathway. Biochem Pharmacol.

[22] Zhang HZ, Kasibhatla S, Wang Y, Herich J, Guastella J, Tseng B, Drewe J, Cai SX (2004). Discovery, characterization and SAR of gambogic acid as a potent apoptosis inducer by a HTS assay. Bioorg Med Chem.

[23] Lu Z, Lu N, Li C, Li F, Zhao K, Lin B, Guo Q (2012). Oroxylin A inhibits matrix metalloproteinase-2/9 expression and activation by up-regulating tissue inhibitor of metalloproteinase-2 and suppressing the ERK1/2 signaling pathway. Toxicology letters.

[24] Yao J, Hu R, Sun J, Lin B, Zhao L, Sha Y, Zhu B, You QD, Yan T, Guo QL (2012). Oroxylin A prevents inflammation-related tumor through down-regulation of inflammatory gene expression by inhibiting NF-kappaB signaling. Mol Carcinog.

[25] Onn A, Isobe T, Itasaka S, Wu W, O'Reilly MS, Ki Hong W, Fidler IJ, Herbst RS (2003). Development of an orthotopic model to study the biology and therapy of primary human lung cancer in nude mice. Clinical cancer research.

[26] Wang H, Zhao L, Zhu LT, Wang Y, Pan D, Yao J, You QD, Guo QL (2014). Wogonin reverses hypoxia resistance of human colon cancer HCT116 cells via downregulation of HIF-1alpha and glycolysis, by inhibiting PI3K/Akt signaling pathway. Molecular carcinogenesis.

[27] Borthwick LA, Gardner A, De Soyza A, Mann DA, Fisher AJ (2012). Transforming Growth Factor-beta1 (TGF-beta1) Driven Epithelial to Mesenchymal Transition (EMT) is Accentuated by Tumour Necrosis Factor alpha (TNFalpha) via Crosstalk Between the SMAD and NF-kappaB Pathways. Cancer microenvironment.

[28] Xie L, Law BK, Chytil AM, Brown KA, Aakre ME, Moses HL (2004). Activation of the Erk pathway is required for TGF-beta1-induced EMT *in vitro*. Neoplasia.

[29] Wei JL, Li ZR, Chen WF, Ma CY, Zhan F, Wu W, Peng YM (2013). AEG-1 participates in TGF-beta1-induced EMT through p38 MAPK activation. Cell biology international.

[30] Sahai E (2007). Illuminating the metastatic process. Nat Rev Cancer.

[31] Brabek J, Mierke CT, Rosel D, Vesely P, Fabry B (2010). The role of the tissue microenvironment in the regulation of cancer cell motility and invasion. Cell communication and signaling.

[32] Bissell MJ, Labarge MA (2005). Context, tissue plasticity, and cancer: are tumor stem cells also regulated by the microenvironment?. Cancer Cell.

[33] Drabsch Y, ten Dijke P (2012). TGF-beta signalling and its role in cancer progression and metastasis. Cancer Metastasis Rev.

[34] Eckert MA, Yang J (2011). Targeting invadopodia to block breast cancer metastasis. Oncotarget.

[35] Wu Y, Zhou BP (2010). TNF-alpha/NF-kappaB/Snail pathway in cancer cell migration and invasion. Br J Cancer.

[36] Min C, Eddy SF, Sherr DH, Sonenshein GE (2008). NF-kappaB and epithelial to mesenchymal transition of cancer. J Cell Biochem.

[37] Zhang YE (2009). Non-Smad pathways in TGF-beta signaling. Cell Res.

[38] Li R, Wang Y, Liu Y, Chen Q, Fu W, Wang H, Cai H, Peng W, Zhang X (2013). Curcumin inhibits transforming growth factor-beta1-induced EMT via PPARgamma pathway, not Smad pathway in renal tubular epithelial cells. PloS one.

[39] Lotz-Jenne C, Luthi U, Ackerknecht S, Lehembre F, Fink T, Stritt M, Wirth M, Pavan S, Bill R, Regenass U, Christofori G, Meyer-Schaller N (2016). A high-content EMT screen identifies multiple receptor tyrosine kinase inhibitors with activity on TGFbeta receptor. Oncotarget.

[40] Pandey MK, Sung B, Ahn KS, Kunnumakkara AB, Chaturvedi MM, Aggarwal BB (2007). Gambogic acid, a novel ligand for transferrin receptor, potentiates TNF-induced apoptosis through modulation of the nuclear factor-kappaB signaling pathway. Blood.

[41] Qi Q, Lu N, Li C, Zhao J, Liu W, You Q, Guo Q (2014). Involvement of RECK in gambogic acid induced anti-invasive effect in A549 human lung carcinoma cells. Mol Carcinog.

