# Supplementary material for: Gambogic acid suppresses cancer invasion and migration by inhibiting TGFβ1-induced epithelial-to-mesenchymal transition
Source: Oncotarget. 2017 Feb 17;8(16):27120–36. doi: 10.18632/oncotarget.15449 (PMC5432322; doi:10.18632/oncotarget.15449)
Supplement: Supplementary file 1 [file oncotarget-08-27120-s001.pdf]

Gambogic acid suppresses cancer invasion and migration by inhibiting TGFβ1-induced epithelial-to-mesenchymal transition

SUPPLEMENTARY FIGURE

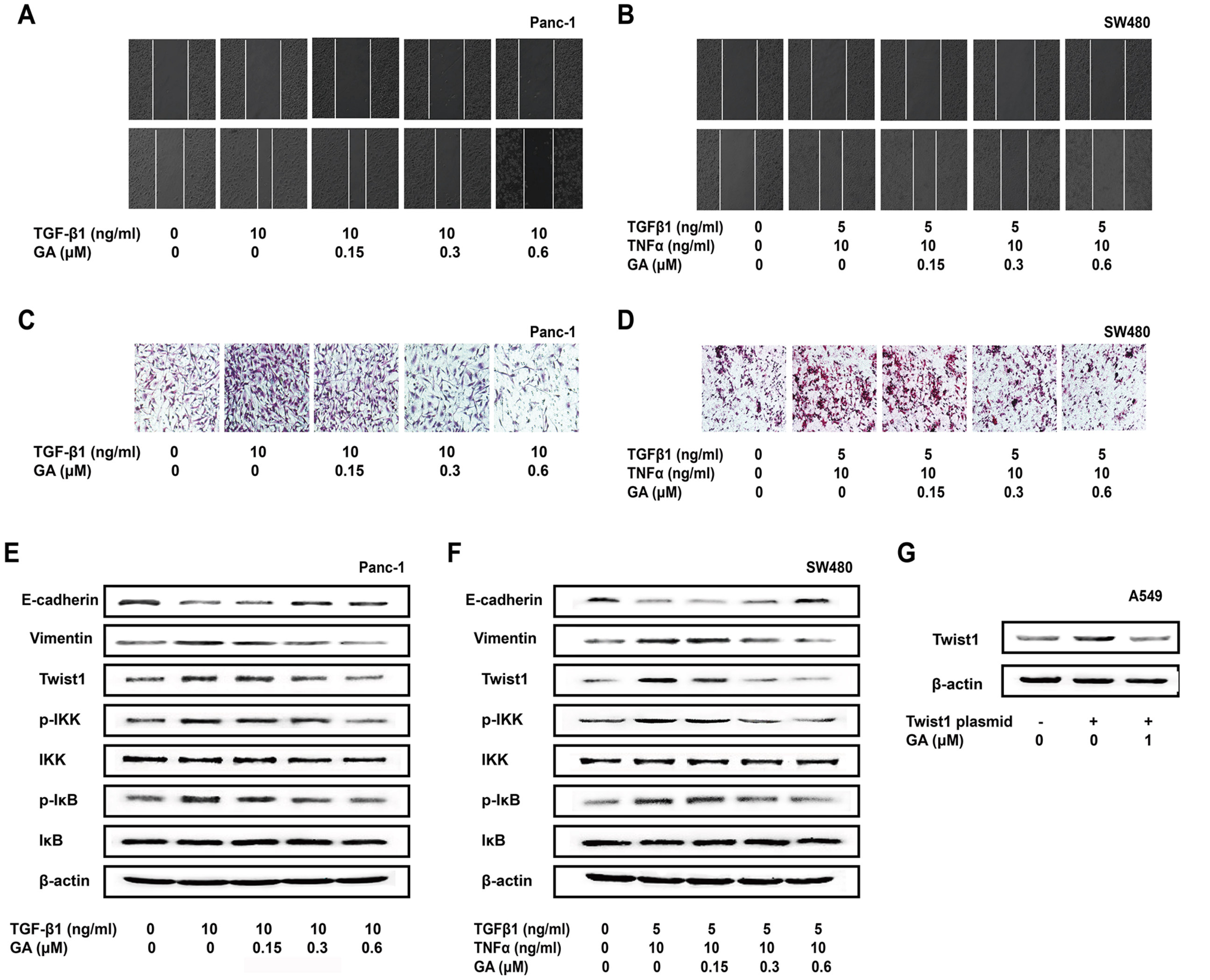

Supplementary Figure 1: GA inhibits migration, invasion and EMT process in Panc-1 and SW480 cells. Panc-1 and SW480 cells were treated with the indicated concentrations of GA, TGFβ1 and TNFα for 24 h. **(A-B)** The effect of GA on the migrated cells were assessed (image magnification: 100×). **(C-D)** The effect of GA on the invaded cells was assessed (image magnification: 200×). **(E-F)** The expression of E-cadherin, vimentin, Twist1, p-IκB, IκB, p-IKK and IKK in the cells was analyzed by western blotting using specific antibodies. An anti-β-actin antibody was used to check equivalent protein loading. **(G)** A549 cells were transfected with TWIST1 plasmids. The effect of GA on the overexpression of TWIST1 was tested. Each experiment was performed at least three times. *$p < 0.05$ compared with the TGFβ1-treated group; **$p < 0.01$ compared with the TGFβ1-treated group.
